# Utility of red dichromatic imaging for identifying the bleeding point in endoscopic hemostasis of colonic diverticular bleeding

**DOI:** 10.1016/j.vgie.2022.01.002

**Published:** 2022-03-14

**Authors:** Soma Fukuda, Taku Sakamoto, Hideo Suzuki, Toshiaki Narasaka, Kiichiro Tsuchiya

**Affiliations:** Department of Gastroenterology, Faculty of Medicine, University of Tsukuba, Tsukuba, Japan

**Keywords:** CDB, colonic diverticular bleeding, RDI, red dichromatic imaging, WLI, white-light imaging

## Abstract

Video 1 Endoscopic hemostasis of colonic diverticular bleeding using red dichromatic imaging to identify the bleeding point.

Endoscopic hemostasis of colonic diverticular bleeding using red dichromatic imaging to identify the bleeding point.

An 82-year-old woman was admitted to our hospital because of hematochezia without pain. She had been admitted to our hospital several times in the previous year for colonic diverticular bleeding (CDB) and had already undergone multiple hemostatic treatments for the CDB. A previous plain computed tomography scan had showed multiple colonic diverticula in the ascending colon and sigmoid colon (Fig. 1), so recurrent colonic diverticular hemorrhage was the primary differential diagnosis.

**Figure 1:**
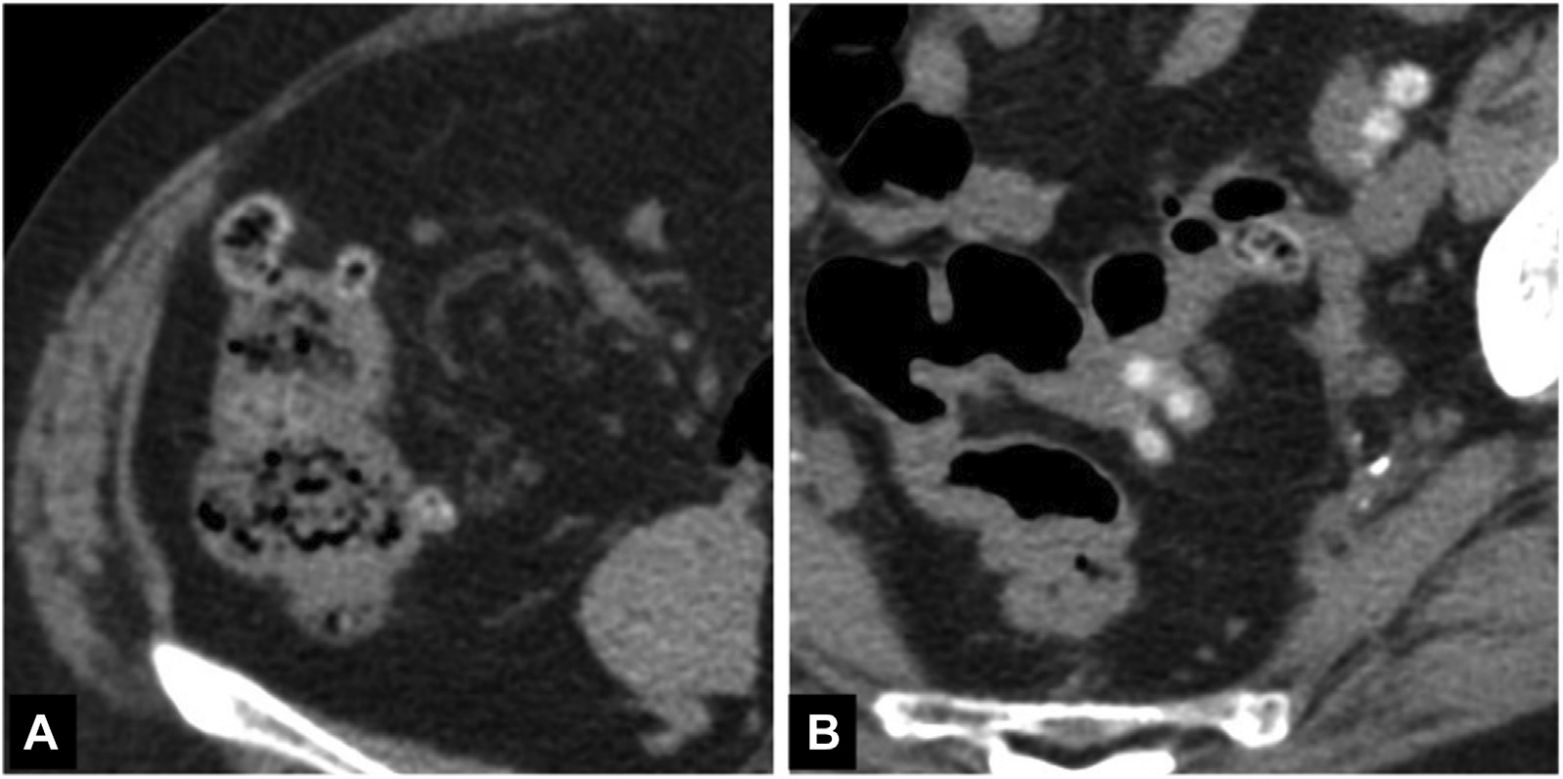
Plain computed tomography scan images. **A,** Diverticula in the ascending colon. **B,** Diverticula in the sigmoid colon.

Urgent colonoscopy was carried out after bowel preparation with polyethylene glycol using the PCF-H290TI colonoscope (Olympus, Tokyo, Japan) and novel EVIS X1 device (Olympus), which is equipped with the red dichromatic imaging (RDI) mode (designed to enhance the visibility of deep blood vessels and bleeding sources) (Video 1, available online at www.VideoGIE.org). White-light imaging (WLI) showed that fresh blood was pooled and oozing from the site of numerous diverticula in the sigmoid colon (Fig. 2). Hemostasis was required, but the exact bleeding point was obscured.

**Figure 2:**
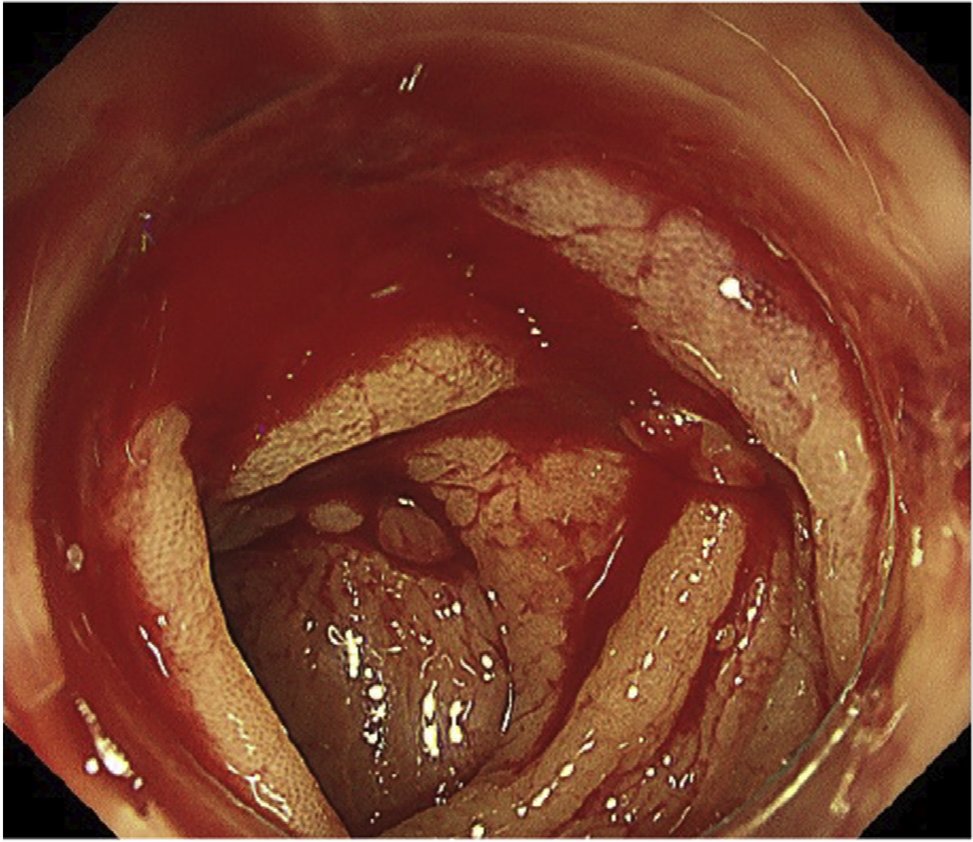
White-light imaging showing active bleeding in the sigmoid colon without clear visualization of the bleeding point.

Once the endoscopic image was switched from WLI to RDI, the blood stream appeared to be a darker yellow as compared with the surrounding blood and was clearly visualized. Immediately, we saw blood oozing from a small diverticulum, and the bleeding point was detected (Fig. 3). The visibility of the bleeding point was better with RDI than with WLI because of the highlighting with shades of color (Fig. 4). The diverticulum was then suctioned into the transparent hood on the endoscope tip (Fig. 5), and hemostasis with endoscopic band ligation was successfully achieved (Fig. 6). The patient was discharged 7 days later, and no rebleeding or adverse events occurred in over 30 days after treatment.

**Figure 3:**
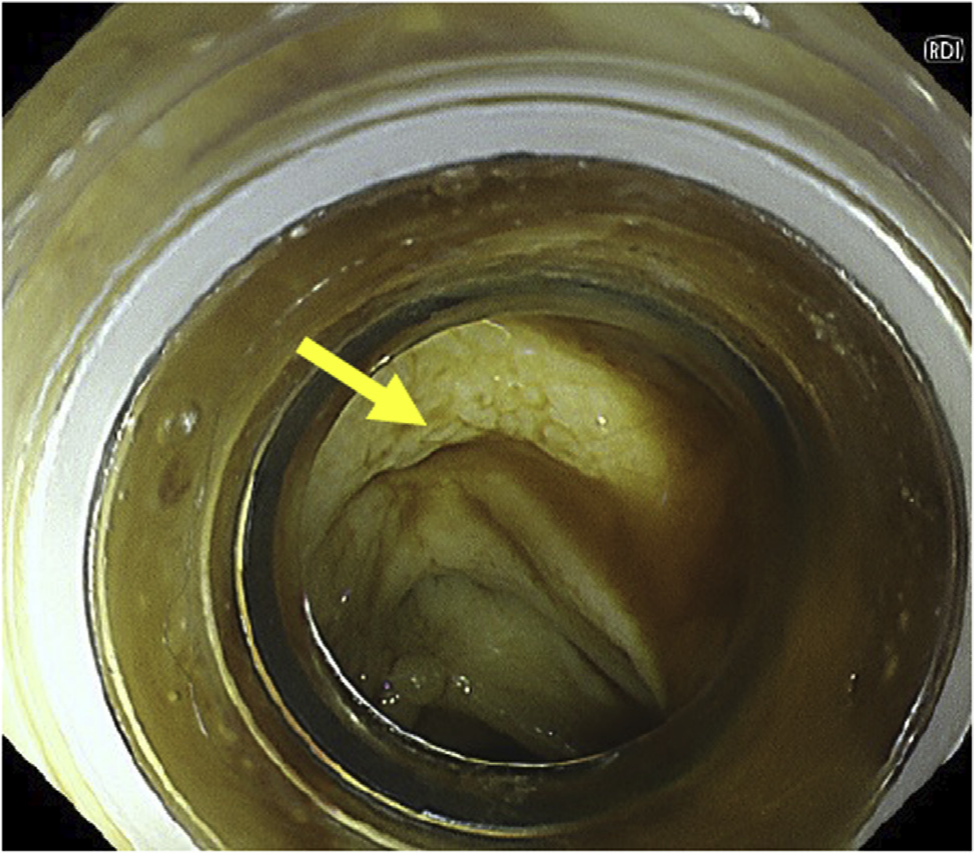
Red dichromatic imaging mode showing blood oozing from the small diverticulum (*arrow*).

**Figure 4:**
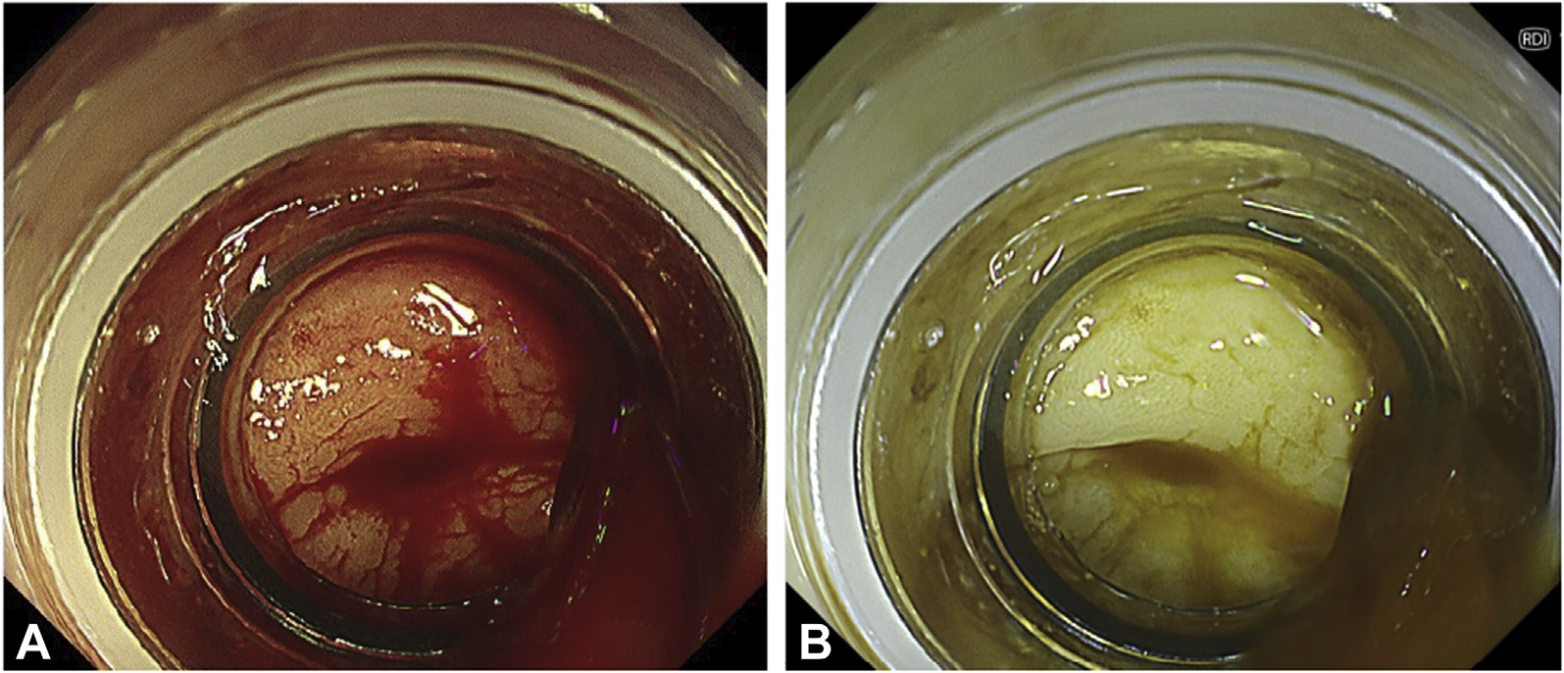
Red dichromatic imaging **(A)** visualizing the blood flow more clearly than white-light imaging **(B)**.

**Figure 5:**
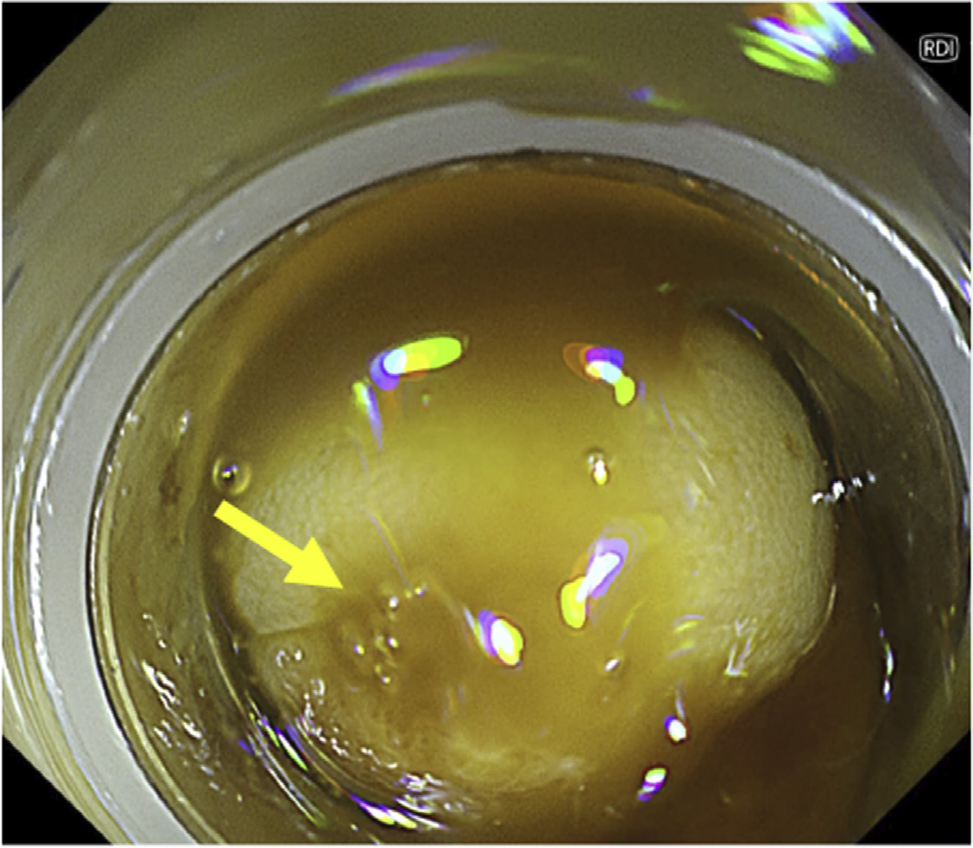
Endoscopic view showing the diverticulum with bleeding visible vessel (*arrow*) suctioned into the transparent hood.

**Figure 6:**
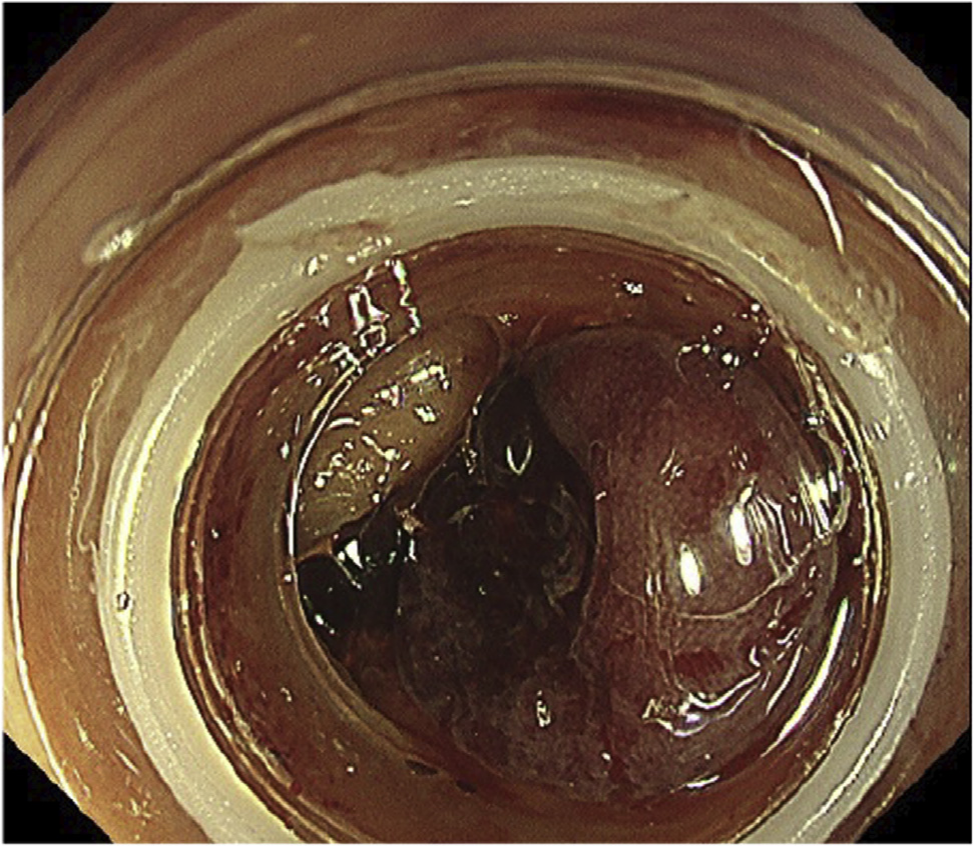
Endoscopic view showing the diverticulum ligated by the band.

CDB is the most common cause of lower GI bleeding in Japan, accounting for 63.6% of all lower GI bleeding cases.^1^ Although the bleeding in 70% to 90% of CDB cases stops spontaneously, rebleeding often occurs in the short term, and in many cases, the responsible diverticulum is the same as that for the previous bleeding.^2^, ^3^, ^4^ Therefore, ligation methods that can completely eliminate the target diverticulum, such as endoscopic band ligation and endoscopic detachable snare ligation, have been reported to be useful for endoscopic hemostasis of CDB.^5^^,^^6^ However, even if active bleeding occurs during urgent colonoscopy, the bleeding point cannot be easily visualized when blood is pooled in the colon. Furthermore, the bleeding often stops spontaneously during examination, and the bleeding diverticulum may not be found. To make matters worse, diverticula are often numerous in the surrounding area, making it difficult to accurately identify the exact source of the bleeding and perform hemostasis. Therefore, it is important to detect the bleeding diverticula quickly and precisely when active bleeding is occurring.

RDI is a novel image-enhanced endoscopic technology that uses 3 types of wavelengths: green (540 nm), amber (600 nm), and red (630 nm), which enhance the visibility of blood vessels in deep tissue.^7^ Amber light is more easily absorbed by the hemoglobin in blood than is red light. This characteristic makes it easier to detect the bleeding point and its peripheral areas with different colors, thus improving quick and safe hemostasis during bleeding while RDI is being performed. Practically, it has been reported that RDI is useful for identifying the source of bleeding in endoscopic hemostasis during endoscopic submucosal dissection or upper GI bleeding.^8^, ^9^, ^10^ In addition, amber light may reduce the psychological stress of physicians during hemostatic procedures: Amber brings out feelings of optimism and confidence, whereas red brings out alarm and irritation for the endoscopist.^11^ For all of these reasons, RDI has the potential to help patients who have recurrent CDB.

This case demonstrates the usefulness of RDI for identifying the bleeding source in colonic diverticular bleeding.

## Disclosure

*All authors disclosed no financial relationships.*

## Supplementary data

Video 1 Endoscopic hemostasis of colonic diverticular bleeding using red dichromatic imaging to identify the bleeding point.
